# Associations of Relationship Experiences, Dating Violence, Sexual Harassment, and Assault With Alcohol Use Among Sexual and Gender Minority Adolescents

**DOI:** 10.1177/08862605211001469

**Published:** 2021-03-14

**Authors:** W. J. Kiekens, L. Baams, J. N. Fish, R. J. Watson

**Affiliations:** 1 University of Groningen, The Netherlands; 2 University of Maryland, College Park, USA; 3 University of Connecticut, Storrs, USA

**Keywords:** sexual minority, gender minority, dating violence, alcohol use, adolescents

## Abstract

Sexual and gender minority (SGM) adolescents report higher rates of dating violence victimization compared with their heterosexual and cisgender peers. Research on dating violence often neglects diversity in sexual and gender identities and is limited to experiences in relationships. Further, given that dating violence and alcohol use are comorbid, research on experiences of dating violence could provide insights into alcohol use disparities among SGM adolescents. We aimed to map patterns of relationship experiences, sexual and physical dating violence, and sexual and physical assault and explored differences in these experiences among SGM adolescents. Further, we examined how these patterns explained alcohol use. We used a U.S. non-probability national web-based survey administered to 13–17-year-old SGM adolescents (*N* = 12,534). Using latent class analyses, four patterns were identified: low relationship experience, dating violence and harassment and assault (72.0%), intermediate dating experiences, sexual harassment, and assault and low levels of dating violence (13.1%), high dating experiences, dating violence, and sexual assault (8.6%), and high dating experiences, dating violence, and sexual harassment and assault (6.3%). Compared to lesbian and gay adolescents, bisexual adolescents reported more experiences with dating, dating violence, and sexual assault, whereas heterosexual adolescents reported fewer experiences with dating, dating violence, and sexual harassment and assault. Compared to cisgender boys, cisgender girls, transgender boys, and non-binary/assigned male at birth adolescents were more likely to experience dating violence inside and outside of relationship contexts. Experiences of dating, dating violence, and sexual harassment and assault were associated with both drinking frequency and heavy episodic drinking. Together, the findings emphasize the relevance of relationship experiences when studying dating violence and how dating violence and sexual harassment and assault might explain disparities in alcohol use.

## Introduction

Sexual and physical dating violence is recognized as a public health problem by the Centers for Disease Control and Prevention (CDC, 2014) and is related to risk behaviors, such as alcohol use (Kaukinen, 2014; Temple et al., 2013). Sexual and gender minority (SGM) adolescents report higher rates of sexual and physical dating violence victimization than their heterosexual and cisgender peers (Blais et al., 2015; Hardesty & Ogolsky, 2020; Martin-Storey et al., 2020; Miller et al., 2016). However, research on dating violence is often limited to experiences in romantic relationships, which oftentimes does not capture experiences with sexual harassment and assault outside of romantic relationships. Further, because dating violence is a potential risk factor for alcohol use (Kidd et al., 2018), examining these experiences among SGM adolescents could provide insights into known alcohol use disparities among and between SGM adolescents (Mereish, 2019). In this study, we explored differences in patterns of dating experiences, sexual and physical dating violence, and experiences of sexual harassment and assault among SGM adolescents. Further, we examined whether these patterns explained differences in alcohol use between SGM adolescents.

## Romantic Relationships and Violence Among SGM Adolescents

Dating and sexual relationships during adolescence are recognized as a normative task (Tolman & McClelland, 2011). Sexual behaviors might be explored in, as well as out of, committed relational contexts—for example, in one-on-one dating, hook-ups, sexting, committed monogamous relationships, or forms of committed non-monogamous relationships (Hammack et al., 2019; Rice et al., 2018; Watson et al., 2018). At the same time, adolescence is a period during which many adolescents experience sexual or physical dating violence, which are two types of intimate partner violence (CDC, 2014), which is generally understood as “physical violence, sexual violence, stalking and psychological aggression (including coercive tactics) by a current or former intimate partner” (Breiding et al., 2015, p. 11).

Compared with their heterosexual and cisgender peers, SGM adolescents are more likely to experience sexual and physical dating violence (Martin-Storey et al., 2020). Despite the value of existing studies comparing SGM adolescents to heterosexual or cisgender adolescents, this work is often framed through a heteronormative lens. That is, historically, research on dating violence has been gendered, with men often being portrayed as the perpetrators and women as the victims (Baker et al., 2013; Blais et al., 2015). Such heteronormative assumptions do not characterize actual experiences in same-sex/gender relationships, relationships between people with different sexual orientations, or relationships with one or more gender non-conforming or transgender partners. Therefore, research among SGM adolescents is needed to better understand experiences with sexual and physical dating violence in this population, and vulnerabilities for certain SGM subgroups.

Studies have identified sexual identity-based differences in sexual and physical dating violence. For example, among sexual minority adolescents bisexual girls reported more sexual and physical dating violence than bisexual boys, as did boys unsure of their sexual identity compared with girls unsure of their sexual identity (Martin-Storey, 2015). A different study found no differences in dating violence between bisexual-and “other”-identifying adolescents compared with their lesbian and gay peers (Langenderfer-Magruder et al., 2016). In a study among high school students, boys questioning their sexual identity reported higher rates of physical dating violence compared with bisexual boys and reported higher rates of sexual dating violence when compared to lesbian girls, gay boys, and bisexual boys (Edwards, 2018). In addition, bisexual adolescents and youth unsure of their sexual identity were more likely to report sexual dating violence when compared to gay and lesbian adolescents (Whitton, Newcomb, et al., 2019).

Adolescent dating violence research is mixed with regard to gender-based differences. Studies using general school-based samples show a higher prevalence of dating violence among cisgender girls compared with boys (Dank et al., 2014), some find the opposite (Martin-Storey, 2015), and others find no clear differences (Edwards, 2018). Further, studies suggest that transgender adolescents experience higher rates of dating violence than their cisgender peers (Dank et al., 2014; Martin-Storey et al., 2020; Mitchell et al., 2014; Whitton, Newcomb, et al., 2019). None of these studies, however, assessed diverse gender identities (e.g., transgender genderqueer, gender non-binary) and whether these groups differed in terms of their experiences with sexual and physical dating violence. Thus, dating violence research should take in to account diverse gender identities, especially considering that some research has reported gender identity-based differences in (dating) violence victimization (Sterzing et al., 2019).

In addition to acknowledging diverse sexual and gender identities, it is important to consider experiences with sexual harassment and assault that might occur outside of dating relationships. Research on sexual and physical dating violence often restricts their samples to participants who have these experiences within romantic relationships (Dank et al., 2014; Edwards, 2018; Johnson et al., 2016; Langenderfer-Magruder et al., 2016; Martin-Storey, 2015). However, given the wide variety of relational configurations SGM adolescents might engage in (e.g., committed non-monogamous relationships, Hammack et al., 2019; Rice et al., 2018; Watson et al., 2018), experiences with violence such as sexual harassment and assault can also occur outside of traditional committed and monogamous relationships (Breiding et al., 2015). Sexual assault is understood as any (attempt of a) sexual act against a person using coercion regardless of the relationship between the victim and the perpetrator (WHO, 2016). Sexual harassment is understood as unwanted sexual contact or non-contact experiences, regardless of the relationship between the victim and the perpetrator (Breiding et al., 2015). Thus, research should consider dating experiences and experiences of sexual harassment and assault to get a broader understanding of SGM adolescents’ dating experiences.

## Sexual and Physical Dating Violence and Alcohol Use

SGM adolescents report higher rates of alcohol use than their heterosexual and cisgender peers (Mereish, 2019), with substantial within-group differences in rates of alcohol use. For example, bisexual adolescents, and especially bisexual females, report high rates of alcohol use compared with heterosexual adolescents than do lesbian and gay adolescents (Marshal et al., 2008; Philips et al., 2017), as well when directly compared to lesbian and gay adolescents (Kerr et al., 2014). For gender identity, studies among sexual minority youth suggest higher alcohol use of cisgender sexual minority boys compared with girls (Watson et al., 2020). Research comparing transgender and cisgender youth finds greater risks of alcohol use for transgender adolescents (Day et al., 2017; De Pedro et al., 2017; Fuxman et al., 2020; Reisner et al., 2015). No consistent subgroup differences are found in alcohol use disparities between gender-minority youth (Rimes et al., 2019; Watson et al., 2020). Moreover, research focusing on identifying the mechanisms driving these sexual identity and gender-based disparities is scarce (Mereish, 2019). Taken together, the current body of research is limited in its understanding of alcohol use disparities between SGM youth, and, more generally, could benefit from research studying mechanisms driving these disparities.

In the context of alcohol use disparities among SGM adolescents, dating violence plays an important role. Although research has argued for alcohol use as risk factor for experiencing dating violence (Spencer et al., 2020), a recent systematic literature review has pointed to dating violence as a risk factor for alcohol use among young men who have sex with men (Kidd et al., 2018). However, studies examining the relation between dating violence and alcohol use among SGM adolescents are scarce and limited in scope. One exception is a study in a community sample of SGM youth that found that sexual and physical dating violence victims reported higher alcohol use compared with non-victims (Langenderfer-Magruder et al., 2016). However, the study was unable to assess differences in these experiences across different sexual and gender identities. Such research is needed to understand and potentially prevent alcohol use disparities among SGM adolescents. In sum, there is limited research on dating violence as an explanation for alcohol use disparities among SGM adolescents.

## The Present Study

The aims of the current study were twofold. First, we aimed to estimate how patterns of relationship experiences, sexual and physical dating violence, and sexual harassment and assault varied across different groups of adolescents on the basis of sexual and gender identity. Second, we aimed to examine how patterns of dating experiences, sexual and physical dating violence, and sexual harassment and assault were associated with alcohol use, and as a potential mediator of alcohol use differences between sexual and gender identities. Given the exploratory nature of this study, no hypotheses regarding patterns of dating experiences, sexual and physical dating violence, and sexual harassment and assault or at risk SGM adolescents are formulated. However, we did expect that patterns characterized by experiences with sexual and physical dating violence, and sexual harassment and assault would be associated with higher alcohol use compared with patterns without sexual and physical dating violence and sexual harassment and assault experiences.

## Methods

### Participants

Data are from the *LGBTQ National Teen Survey*, a U.S. non-probability national web-based survey administered to 13–17-year-old English speaking SGM adolescents (Watson et al., 2020). The data were collected between April and December 2017 in partnership with the Humans Rights Campaign (HRC). The aim of the larger study was to gain a better understanding of victimization, school experiences, health behaviors, and family relationships of SGM adolescents. Participants were recruited via social media in various ways. Influencers and the HRC shared the survey link via their social media profiles. Further, HRC partner organizations spread the survey link via email or direct communication. As a reward, participants were offered HRC wristbands and could enter a random drawing of $50 gift cards. The IRB of the University of Connecticut approved the study protocol.

Of all participants who started the survey, 17,112 were eligible for the larger study (i.e., 13–17 years old, reside inside the United States, and self-identified as SGM), completed more than 10% of the survey questions, and were deemed to have provided valid responses to survey questions (for procedures, refer to Watson et al., 2020). Data from participants were deleted if they had missing data on all items assessing relationship status and sexual and physical dating violence, yielding a final dataset of *N* = 12,534. Missingness on alcohol use variables was 4.2% and missingness on covariates (race/ethnicity, region, and living situation) ranged from .1 to 2.0%. Descriptive statistics are presented in Table 1.

**Table 1.** Descriptive Statistics.

Variable	Percentage
Sexual identity	
Gay and lesbian	36.7
Bisexual	34.2
Heterosexual	1.59
Queer	4.4
Pansexual	13.8
Asexual	4.8
Questioning	2.5
Other	2.2
Gender identity	
Cisgender boy	21.9
Cisgender girl	43.6
Transgender boy	8.5
Transgender girl	1.1
Non-binary/assigned male at birth	22.5
Non-binary/assigned female at birth	2.5
Relationship experiences	
Never dated	31.4
Dated in the past	43.7
Dating, not exclusive	5.6
Dating, exclusive	18.4
Multiple partners	1.1
Sexual DV in relationship context	
0 times	87.1
1 time	5.6
2 or 3 times	4.6
4 or 5 times	1.3
6 times or more	1.4
Physical DV in relationship context	
0 times	92.8
1 time	3.2
2 or 3 times	2.4
4 or 5 times	.7
6 times or more	.9
General sexual assault	
0 times	79.8
1 time	8.9
2 or 3 times	7.6
4 or 5 times	1.9
6 times or more	1.8
General sexual harassment	.83 (.74)*
Alcohol use frequency	
Yes	27.1
Binge drinking	
Yes	9.7
Region	
Northeast	18.1
Midwest	23.5
South	23.6
West	21.9
Race/ethnicity	
White	64.7
Black/African American	4.9
Native American	.5
Asian American	3.9
Hispanic/Latino	10.5
Bi/multiracial	13.8
Other	1.8

*Note*. *Mean and standard deviation are given.

### Measures

#### Alcohol use.

Three items were used to measure alcohol use (Kann et al., 2016). The first item was “During your life, on how many days have you had at least one drink of alcohol” with response options ranging from 0 = *0 days* to 6 = *100 days or more*. Those who reported alcohol use were prompted with two additional questions: “During the past 30 days, on how many days did you have at least one drink of alcohol?” to assess alcohol use frequency and “During the past 30 days, on how many days did you have 5 or more drinks of alcohol in a row, that is, within a couple of hours?” to assess heavy episodic drinking. Response options were 0 = *0 days*, 1 = *1 or 2 days*, 2 = *3 to 5 days*, 3 = *6 to 9 days*, 4 = *10 to 19 days*, 5 = *20 to 29 days*, and 6 = *All 30 days*. Participants who reported no lifetime alcohol use were coded as having 0 drinks in the past 30 days and 0 heavy episodic drinking episodes in the past 30 days. Because both alcohol use frequency and heavy episodic drinking were skewed and represent serious health risks to adolescents, we dichotomized both items. Answers for alcohol use frequency were recoded such that they reflected 0 = *No drinking in the past 30 days* and 1 = *Any drinking in the past 30 days*, and for heavy episodic drinking answers were recoded as 0 = *No heavy episodic drinking in the past 30 days* and 1 = *Any* heavy *episodic drinking in the past 30 days*.

#### Relationship experiences.

To assess experiences with romantic relationships, the following item was used: “Which of the following best describes your current dating situation?” Answer options were 0 = *I have never dated*, 1 = *I am not dating anyone now, but I have in the past*, 2 = *I am dating someone but we are not exclusive*, 3 = *I am in an exclusive dating relationship with one person*, and 4 = *I am dating multiple people now*.

#### Sexual and physical dating violence.

Two variables were used to assess sexual and physical dating violence experiences in the past 12 months. The first variable assessed *sexual dating violence* and participants were asked “During the past 12 months, how many times did someone you were dating or going out with force you to do sexual things that you did not want to do? (Count such things as kissing, touching, or being physically forced to have sexual intercourse.)” (Kann et al., 2016). The second variable assessed *physical dating violence:* “During the past 12 months, how many times did someone you were dating or going out with physically hurt you on purpose? (Count such things as being hit, slammed into something, or injured with an object or weapon.)” (Kann et al., 2016).

#### Sexual harassment and assault.

Two variables were used to assess sexual harassment and assault. The first variable assessed *sexual assault:* “During the past 12 months, how many times did anyone force you to do sexual things that you did not want to do? (Count such things as kissing, touching, or being physically forced to have sexual intercourse.)” (Kann et al., 2016). For these three items, answer options were 0 = *0 times*, 1 = *1 time*, 2 = *2 or 3 times*, 3 = *4 or 5 times*, or 4 = *6 or more times*. To assess *sexual harassment* in the past 12 months, a continuous variable based on the mean score of five items was constructed (Hill & Kearl, 2011; *α* = .78). All questions captured experiences that were sexual in nature. An example question read “During the past 12 months, how many times did someone make unwelcome sexual comments, jokes, or gestures to or about you” with answer options 0 = *0 times*, 1 = *1 time*, 2 = *2 or 3 times*, 3 = *4 or 5 times*, or 4 = *6 or more times.*

#### Sexual identity.

To assess sexual identity, the following item was used “How do you describe your sexual identity?” Answer categories were 1 = *Gay or lesbian*, 2 = *Bisexual*, 3 = *Straight, that is not gay*, and 4 = *Something else*. Participants who chose *something else* were prompted with the question “By something else, do you mean that” with answer option 5 = *Queer*, 6 = *Pansexual*, 7 = *Asexual*, 8 = *Questioning*, and 9 = *Other, (please state)*. These two items were combined into one sexual identity measure, in which Gay or lesbian was used as the reference category.

#### Gender identity.

Two items were used to assess gender identity. The first item was “What is your current gender identity? Please select all that apply.” with answer categories 1 = *Male*, 2 = *Female*, 3 = *Trans male/Trans boy*, 4* = Trans female/Trans girl*, 5 = *Non-binary*, 6* = Genderqueer/Gender non-conform*, and 7 = *Different identity (please state)*. The second item was “What sex were you assigned at birth?” with 1 = *Male* and 2 = *Female*. Gender identity was coded as *cisgender boy* or *cisgender girl* if participants had concordant sex assigned at birth and gender identities. If participants reported only binary male identities (i.e., *male* or *trans male/trans boy*) for their gender identity and were assigned female at birth, gender identity was coded as *transgender boy*. If participants reported only binary female identities (i.e., *female* or *trans female/trans girl*) for gender identity and were assigned male at birth, gender identity was coded as *transgender girl*. For participants who reported a non-binary and/or genderqueer/non-conforming identity and assigned male or female at birth, gender identity was coded as non-binary/assigned male at birth or *non-binary/assigned female at birth*. Cisgender boy was used as the reference category in our analyses.

#### Race/ethnicity.

For race/ethnicity the following item was used “How would you describe yourself? (select all that apply).” Answer categories were 1 = *White, non-Hispanic, non-Latino*, 2 = *Black or African American*, 3 = *American Indian or Alaska Native*, 4 = *Asian or Pacific Islander*, 5 = *Latino, Hispanic, or Mexican American*, 6* = Other (please state)*. Data were recoded as 1 = *White*, 2 = *Black*, 3 = *Native American*, 4 = *Asian American*, 5 = *Hispanic/Latino*, 6 = *Bi/multiracial*, and 7 = *Other*.

#### Region.

The state in which participants lived was assessed with one item. Based on this, four regions were distinguished, namely 1 = *Northeast*, 2 = *Midwest*, 3 = *South*, and 4 = *West*.

#### Living situation.

To assess living situation, the following item was used: “With whom do you currently live? (Check all that apply)” in order to assess their living situation. Response options were 1 = *Alone*, 2 = *Mother(s)*, 3 = *Father(s)*, 4 = *Adoptive mother(s)*, 5 = *Adoptive father(s)*, 6 = *Siblings*, 7 = *Lover/partner*, 8* = Friend(s)*, 9 = *Grandparent(s)*, 10 = *Uncle(s)/aunt(s)*, 11 = *Stepparents(s)*, 12 = *Foster parent(s)*, 13 = *Other parent*, 14 = *Group home*, 15 = *Homeless (no fixed address)*, and 16 = *Other*. Participants reported living with their biological, adoptive, or stepparents were coded as 0, those who did not live with their biological, adoptive, or stepparents were coded as 1.

### Analytic Strategy

Latent class analysis (LCA) was used to estimate patterns of dating status, sexual and physical dating violence, and sexual harassment and assault in Mplus version 8.3 (Muthén & Muthén, 1998–2017). Dating violence and sexual assault were treated as ordinal, sexual harassment as continuous, and the dating experiences were treated as a nominal variable. One-through-six-class models were fit to the data. Model fit was assessed by comparing estimates of relative fit, including log-likelihood, Akaike’s Information Criterion (AIC), and sample-sized adjusted Bayesian Information Criterion (Adj BIC), where lower values indicated better fit to the data. Vuong–Lo–Mendell–Rubin likelihood ratio test (VLMR) and Lo–Mendell–Rubin Likelihood Ratio Test (LMR) were also used to assess fit, where significant *p-*value indicates that current model is a better fit to the data compared to a *k*–1 class model. Entropy, where a value closer to one reflects a better classification of participants, and the interpretability of the classes were also considered when selecting a model.

Next, using the best fitting LCA solution, we assessed the relation between sexual and gender identity and class membership using a 3-step LCA procedure in Mplus (Asparouhov & Muthén, 2014; Feingold et al., 2014). This approach uses a multinomial logistic regression framework to assess the relation between covariates and class membership compared to a reference class and accounts for measurement error when introducing covariates to the model (e.g., sexual and gender identity). Last, indirect effects were estimated to test if class membership explained the association between sexual and gender identity with drinking frequency and heavy episodic drinking in one model using a robust weighted least squares estimator which employs a diagonal weight matrix (ESTIMATOR = WLSMV in Mplus; Muthén et al., 2016). Within the path analyses, a continuous latent response variable underlying the observed dichotomous alcohol use variables was used. Race/ethnicity, region, age, and living situation were used as covariates in all analyses.

## Results

### Relationship and Dating Violence Patterns

One-through-six-class models were tested (Table 2). The log-likelihood, AIC, and Adj BIC decreased with each model, but this decline leveled off from the fourth class onwards. Fit patterns indicated that model fit improvements were smaller when comparing adjacent models over four classes. The VLMR and the LMR supported the four class-class solution because results testing a given model to a *k*–1 class model showed that model fit did not improve in the five and six-class solution. Entropy was highest for the four-class solution as well, indicating that participants were best classified in the four-class solution. Given the clear interpretability of the four-class solution coupled with the fit statistics, a four-class solution was deemed the best-fitting model.

**Table 2.** Fit Statistics for LCAs on Dating Experiences and Sexual and Physical Dating Violence.

	LL	AIC	Adj BIC	Entropy	VLMR	LMR	Min–Max *N*
1 class	−46967.26	93970.52	94047.17	–	–	–	–
2 classes	−42546.04	85164.07	85317.37	.81	<.00	<.00	2,268–10,266
3 classes	−41607.34	83322.69	83552.64	.78	<.00	<.00	856–9,016
4 classes	−41080.62	82305.25	82611.84	.82	<.00	<.00	794–9,017
5 classes	−40727.66	81635.32	82018.57	.79	.83	.83	767–8,081
6 classes	−40479.42	81174.83	81634.73	.80	.76	.76	594–7,861

*Note*. LL = Log-likelihood; AIC = Akaike’s Information Criterion; Adj BIC = Adjusted Bayesian Information Criterion; VLMR = Vuong–Lo–Mendell–Rubin likelihood ratio test; LMR = Lo–Mendell–Rubin likelihood ratio test.

Table 3 displays probabilities of relationships, sexual and physical dating violence, and sexual harassment and assault for all classes. Participants in the largest class (72.0% “Few dating experiences and low dating violence, assault, and harassment”) had the highest probability of having never dated and had low probabilities and mean scores of experiencing sexual or physical dating violence and general sexual harassment and assault. The second class (13.1% “Intermediate exposure to harassment and assault”) consisted of participants who had intermediate probabilities of having dating experiences (especially past dating experiences), low probabilities of sexual and physical dating violence, and an intermediate probability and mean on general sexual harassment and assault. The third class (8.6% “High exposure to dating violence and assault”) had somewhat higher probabilities of dating experiences compared with the previous class and especially higher probabilities of sexual and physical dating violence, and general sexual assault, but lower mean score of general sexual harassment. The fourth class (6.3% “High exposure to dating violence, assault, and harassment”) had similar probabilities of having dating experiences as the third class, but higher probabilities and mean scores of sexual and physical dating violence, and general sexual harassment and assault.

**Table 3.** Probabilities of Relationship Experiences, Sexual and Physical Dating Violence, and Sexual Harassment and Assault Across Four Classes.

	Overall	Few Dating Experiences and Low Dating Violence, Assault, and Harassment	Intermediate Exposure to Harassment and Assault	High Exposure to Dating Violence and Assault	High Exposure to Dating Violence, Assault, and Harassment
	(*n* = 12,534)	(*n* = 9,017)	(*n* = 1,640)	(*n* = 1,083)	(*n* = 794)
Relationship experiences					
Never dated	.31	.40	.22	.01	.07
Dated in the past	.44	.39	.50	.60	.52
Dating, not exclusive	.06	.04	.08	.09	.09
Dating, exclusive	.18	.16	.19	.29	.28
Multiple partners	.01	.00	.02	.01	.04
Sexual DV in relationship context					
0 times	.87	.99	.95	.27	.41
1 time	.06	.01	.02	.37	.13
2 or 3 times	.05	.00	.03	.26	.23
4 or 5 times	.01	.00	.00	.06	.10
6 times or more	.01	.00	.00	.05	.13
Physical DV in relationship context					
0 times	.93	.98	.94	.79	.59
1 time	.03	.01	.03	.11	.14
2 or 3 times	.02	.01	.02	.07	.14
4 or 5 times	.01	.00	.01	.01	.06
6 times or more	.01	.00	.01	.02	.07
General sexual assault					
0 times	.80	.97	.71	.21	.20
1 time	.09	.03	.14	.38	.16
2 or 3 times	.08	.01	.13	.31	.33
4 or 5 times	.02	.00	.02	.07	.14
6 times or more	.02	.00	.01	.05	.17
General sexual harassment	.83 (.74)	.39 (.01)	1.68 (.04)	1.14 (.04)	2.90 (.04)

### Associations of Sexual and Gender Identity With Classes

Table 4 presents the results of the multinomial logistic regression obtained from the 3-step procedure portraying the association of sexual identity and gender identity and class membership. Compared to gay and lesbian adolescents, bisexual adolescents had higher odds of being in the “High exposure to dating violence and assault” class and heterosexual adolescents had lower odds of being in the “High exposure to dating violence, assault, and harassment” class than in the “Few dating experiences and low dating violence, assault, and harassment” class. In the online supplementary analyses with bisexual as reference group are presented.

**Table 4.** Multinomial Regression Analyses With Sexual Identity and Gender Identity Predicting Class Membership.

	Intermediate Exposure to Harassment and Assault^a^		High Exposure to Dating Violence and Assault^a^		High Exposure to Dating Violence, Assault, and Harassment^a^	
	Odds Ratio	95% CI	Odds Ratio	95% CI	Odds Ratio	95% CI
Sexual identity						
Gay and lesbian (ref)						
Bisexual	.89	[.74, 1.05]	1.56**	[1.24, 1.89]	1.22	[.96, 1.48]
Heterosexual	.86	[.33, 1.40]	.93	[.36, 1.51]	.30***	[.00, .64]
Queer	.83	[.51, 1.16]	.78	[.44, 1.12]	.90	[.51, 1.28]
Pansexual	1.20	[.92, 1.47]	1.37	[.99, 1.74]	1.23	[.89, 1.58]
Asexual	1.21	[.81, 1.61]	.91	[.51, 1.30]	1.20	[.72, 1.68]
Questioning	.90	[.49, 1.31]	.65	[.24, 1.07]	.75	[.30, 1.20]
Other	1.13	[.58, 1.68]	.96	[0,40, 1.52]	1.54	[.79, 2.29]
Gender identity						
Cisgender boy (ref)						
Cisgender girl	1.08	[.87, 1.28]	2.34***	[1.62, 3.05]	1.74**	[1.25, 2.22]
Transgender boy	1.07	[.72, 1.43]	4.10***	[2.52, 5.68]	2.76**	[1.74, 3.77]
Transgender girl	1.25	[.41, 2.10]	2.28	[.28, 4.28]	3.07	[.82, 5.32]
Non-binary/assigned male at birth	1.16	[.89, 1.43]	3.80***	[2.54, 5.07]	2.45**	[1.69, 3.21]
Non-binary/assigned female at birth	1.23	[.69, 1.77]	1.75	[.53, 2.96]	1.59	[.65, 2.53]

*Note*. Controlled for race/ethnicity, region, age, and living situation.

^a^Compared with the “Few dating experiences and low dating violence, assault, and harassment” class.

*p < .05. **p < .01. ***p < .001.

Compared with cisgender boys, cisgender girls, transgender boys, and non-binary/assigned male at birth adolescents had higher odds of being in the “High exposure to dating violence and assault” class and the “High exposure to dating violence, assault, and harassment” class than in the “Few dating experiences and low dating violence, assault, and harassment” class. In the online supplementary analyses with cisgender girls, transgender boys, and transgender girls as reference group are presented.

### Associations With Alcohol Use

Table 5 provides an overview of the direct effects of sexual and gender identity on class membership and alcohol use, as well as the direct effects of class membership on alcohol use. Table 6 provides the indirect effects of sexual and gender identity on alcohol use through class membership. The model fit the data well (*χ*^2^ = 598.78, *p* < .01; RMSEA = .01; CFI = .97; SRMR = .08). Focusing on the direct effects of class membership on alcohol use, adolescents in any of the other classes drank more frequently and reported higher rates of heavy episodic drinking than those in the “Few dating experiences and low dating violence, assault, and harassment” class.

**Table 5.** Direct Associations Between Dating Violence Classes and Drinking Frequency and Binge Drinking.

	Intermediate Exposure to Harassment and Assault		High Exposure to Dating Violence and Assault		High Exposure to Dating Violence, Assault, and Harassment		Drinking Frequency		Binge Drinking	
	*b*	95% CI	*b*	95% CI	*b*	95% CI	*b*	95% CI	*b*	95% CI
Sexual identity										
Gay and lesbian (ref)										
Bisexual	−.05	[−.12, .02]	.17***	[.09, .25]	.07	[−.02, .16]	.03	[−.05, .10]	−.01	[−.11, .08]
Heterosexual	−.04	[−.28, .20]	−.01	[−.26, .24]	−.41*	[−.75, −.06]	.18	[−.08, .43]	.60***	[.30, .90]
Queer	−.07	[−.22, .07]	−.10	[−.27, .07]	−.04	[−.22, .14]	.06	[−.08, .21]	.10	[−.10, .30]
Pansexual	.06	[−.04, .15]	.10	[.00, .20]	.07	[−.04, .19]	−.05	[−.14, .05]	−.09	[−.22, .05]
Asexual	.07	[−.07, .21]	−.05	[−.20, .11]	.07	[−.10, .23]	−.53***	[−.68, −.37]	−.57***	[−.82, −.33]
Questioning	−.05	[−.24, .14]	−.16	[−.39, .07]	−.12	[−.37, .13]	−.28**	[−.49, −.08]	−.21	[−.50, .09]
Other	.05	[−.14, .24]	−.05	[−.27, .18]	.20	[−.01, .42]	−.46***	[−.69, −.23]	−.59**	[−.95, −.23]
Gender identity										
Cisgender boy (ref)										
Cisgender girl	.01	[−.07, .09]	.28***	[.19, .38]	.19**	[.08, .29]	−.25***	[−.34, −.17]	−.31***	[−.41, −.20]
Transgender boy	.00	[−.13, .12]	.52***	[.39, .65]	.39***	[.25, .54]	−.30***	[−.43, −.17]	−.41***	[−.59, −.24]
Transgender girl	.08	[−.19, .35]	.27	[−.05, .58]	.42*	[.10, .73]	−.28*	[−.55, .00]	−.24	[−.57, .10]
Non-binary/assigned male at birth	.03	[−.07, .12]	.48***	[.37, .58]	.32***	[.20, .44]	−.37***	[−.47, −.27]	−.51***	[−.65, −.37]
Non-binary/assigned female at birth	.08	[−.10, .26]	.19	[−.05, .42]	.17	[−.07, .41]	−.40***	[−.61, −.19]	−.33*	[−.60, −.07]
Dating violence classes										
Few dating experiences and low dating violence, assault, and harassment										
Intermediate exposure to harassment and assault							.16***	[.12, .20]	.18***	[.13, .24]
High exposure to dating violence and assault							.18***	[.13, .23]	.20***	[.13, .26]
High exposure to dating violence, assault, and harassment							.31***	[.26, .36]	.41***	[.34, .48]

*Note*. Controlled for race/ethnicity, region, age, and living situation.

*p < .05 **p < .01 ***p < .001.

**Table 6.** Indirect Effects of Analyses With Dating Violence Classes Predicting Drinking Frequency and Binge Drinking.

	Drinking Frequency						Binge Drinking					
	Intermediate Exposure to Harassment and Assault		High Exposure to Dating Violence and Assault		High Exposure to Dating Violence, Assault, and Harassment		Intermediate Exposure to Harassment and Assault		High Exposure to Dating Violence and Assault		High Exposure to Dating Violence, Assault, and Harassment	
	*b*	95% CI	*b*	95% CI	*b*	95% CI	*b*	95% CI	*b*	95% CI	*b*	95% CI
Sexual identity												
Gay and lesbian (ref)												
Bisexual	−.01	[−.02, .00]	.03***	[.01, .05]	.02	[−.01, .05]	−.01	[−.02, .00]	.03***	[.01, .05]	.03	[−.01, .06]
Heterosexual	−.01	[−.05, .03]	.00	[−.05, .04]	−.13*	[−.23, −.02]	−.01	[−.05, .04]	.00	[−.05, .05]	−.17*	[−.31, −.02]
Queer	−.01	[−.04, .01]	−.02	[−.05, .01]	−.01	[−.07, .04]	−.01	[−.04, .01]	−.02	[−.05, .01]	−.02	[−.09, .06]
Pansexual	.01	[−.01, .02]	.02	[.00, .03]	.02	[−.01, .06]	.01	[−.01, .03]	.02	[.00, .04]	.03	[−.02, .08]
Asexual	.01	[−.01, .03]	−.01	[−.04, .02]	.02	[−.03, .07]	.01	[−.01, .04]	−.01	[−.04, .02]	.03	[−.04, .09]
Questioning	.01	[−.02, .04]	−.03	[−.07, .01]	−.04	[−.11, .04]	−.01	[−.04, .03]	−.03	[−.08, .02]	−.05	[−.15, .05]
Other	.01	[−.02, .04]	−.01	[−.05, .03]	.06	[.00, .13]	.01	[−.03, .04]	−.01	[−.05, .03]	.08	[−.01, .17]
Gender identity												
Cisgender boy (ref)												
Cisgender girl	.00	[−.01, .02]	.05***	[.03, .07]	.06**	[.02, .09]	.00	[−.01, .02]	.06***	[.03, .08]	.08**	[.03, .12]
Transgender boy	.00	[−.02, .02]	.09***	[.06, .13]	.12***	[.07, .17]	.00	[−.02, .02]	.10***	[.06, .14]	.16***	[.10, .22]
Transgender girl	.01	[−.03, .06]	.05	[−.01, .11]	.13*	[.03, .23]	.02	[−.04, .07]	.05	[−.01, .12]	.17*	[.04, .30]
Non-binary/assigned male at birth	.01	[−.01, .02]	.08***	[.05, .11]	.10***	[.06, .14]	.01	[−.01, .02]	.09***	[.06, .13]	.13***	[.08, .18]
Non-binary/assigned female at birth	.01	[−.02, .04]	.03	[−.01, .07]	.05	[−.02, .13]	.02	[−.02, .05]	.04	[−.01, .08]	.07	[−.03, .17]

*Note*. Controlled for race/ethnicity, region, age, and living situation.

*p < .05 **p < .01 ***p < .001.

Compared to gay and lesbian adolescents, asexual, questioning and adolescents reporting something “other” than what was offered in the survey consumed alcohol less frequently. Compared to gay and lesbian adolescents, heterosexual adolescents had higher rates of heavy episodic drinking, whereas asexual and adolescents reporting “other” sexual identities had lower rates of heavy episodic drinking. Indirect effects indicated that differences in drinking frequency as well as heavy episodic drinking between gay and lesbian adolescents and bisexual adolescents were explained by class membership in the “High exposure to dating violence and assault” class. Differences in drinking frequency as well as heavy episodic drinking between gay and lesbian adolescents and heterosexual adolescents were explained by class membership in the “High exposure to dating violence, assault, and harassment” class.

Last, gender identity comparisons showed that, compared with cisgender boys, cisgender girls, transgender boys, transgender girls (only drinking frequency) non-binary/assigned male at birth adolescents, and non-binary/assigned female at birth adolescents reported less frequent drinking and lower rates of heavy episodic drinking. Indirect effects indicated differences in drinking frequency and heavy episodic drinking, where differences between cisgender boys (reference group) and cisgender girls, transgender boys, and non-binary/assigned male at birth adolescents were explained through class membership in the “High exposure to risk of dating violence and assault” class. Similarly, differences in drinking frequency and heavy episodic drinking between cisgender boys and cisgender girls, transgender boys, transgender girls, and non-binary/assigned male at birth adolescents were explained by class membership in the “High exposure to dating violence, assault, and harassment” class.

## Discussion

In this study, we sought to map patterns of relationship experiences, sexual and physical dating violence, and sexual and physical assault and explored differences in these experiences among SGM adolescents. Further, we examined how these patterns explained alcohol use. Four distinct groups that reflect experiences with relationships, sexual and physical dating violence, and sexual harassment and assault were identified. These findings show the diversity of (sexual) violence experiences among SGM adolescents and underline the importance of considering such experiences that may occur inside and outside relationship contexts.

Compared to lesbian and gay adolescents, we found that bisexual adolescents had more dating experiences, experiences with sexual and physical dating violence, and sexual assault. This is noteworthy given that previous research directly comparing gay and lesbian adolescents with bisexual adolescents has not yet documented differences in dating violence (Edwards, 2018; Langenderfer-Magruder et al., 2016; Whitton, Newcomb, et al., 2019), yet support growing research on the disproportionate health risk for bisexual adolescents (Marshal et al., 2011). A potential explanation for this difference is that we assessed experiences with sexual assault that may not have happened *inside* of a relationship context, something that may be particularly prevalent among bisexual adolescents. Further, compared to lesbian and gay adolescents, heterosexual adolescents had fewer dating experiences, and fewer experiences with physical dating violence, sexual harassment, and assault. Because, in our sample, all heterosexual adolescents identified as a gender minority, this effect can be interpreted as heterosexual gender minorities having a lower risk of physical dating violence, and sexual harassment and assault than lesbian and gay adolescents. Notwithstanding that profound gender identity-based differences in experiencing physical dating violence, and sexual harassment and assault were found.

Overall, cisgender girls had more dating experience and more experiences with sexual and physical dating violence and sexual harassment and assault compared with cisgender boys. This finding is informative to the current body of research which currently present mixed findings of dating violence when comparing cisgender boys and girls (Dank et al., 2014; Edwards, 2018; Martin-Storey, 2015). Further, compared to cisgender boys, transgender boys and non-binary/assigned male at birth adolescents reported more dating experiences and experiences with sexual and physical dating violence and sexual harassment and assault. These results echo previous findings of transgender adolescents reporting higher rates of sexual and physical dating violence compared to cisgender adolescents (Dank et al., 2014; Mitchell et al., 2014; Whitton, Dyar, et al., 2019), and extend these findings to non-binary/assigned male at birth adolescents and to experiences of harassment and assault that may occur outside of dating relationships.

As expected, classes characterized by having more dating experiences, experiences with sexual and physical dating violence, and sexual harassment and assault were associated with drinking frequency and heavy episodic drinking. Thus, regardless of the context of (sexual) violence, we found an association with drinking frequency and heavy episodic drinking.

Finally, patterns of relationship experiences, dating violence, and sexual harassment and assault partially explained differences between sexual and gender identity in drinking frequency and heavy episodic drinking. Having more dating experiences and more experiences with sexual and physical dating violence and sexual assault explained drinking frequency for bisexual adolescents. Similarly, having fewer dating experiences, and lower risk of sexual and physical dating violence, and sexual harassment and assault explained heavy episodic drinking for heterosexual adolescents. Further, having more dating experiences and more experiences with sexual and physical dating violence and sexual harassment and assault were associated with drinking frequency and heavy episodic drinking for cisgender girls, transgender boys, transgender girls, and non-binary/assigned male at birth adolescents.

Taken together, our findings highlight the role that patterns of relationship experiences and sexual and physical dating violence and sexual harassment and assault play in the associations between sexual orientation, gender identity, and alcohol use. Our study provides new insights given that few studies have compared alcohol use among SGM adolescents using such diverse sexual and gender identity labels. Intervention programs on relationships among SGM youth should be aware that bisexual, cisgender sexual minority girls, transgender boys, and non-binary/assigned male at birth youth are more likely to experience dating violence and sexual harassment and assault. Further, our results indicate that these programs cannot only support SGM youth regarding relationships, but also mitigate their alcohol use.

## Limitations and Future Research

Findings from this study should be interpreted with some limitations in mind. First, data in this study come from a cross-sectional non-probability sample of SGM adolescents. This limits generalization of results to the SGM adolescent population. Further, we cannot make inferences about the causal relation between patterns of dating relationships sexual and physical dating violence, and sexual harassment and assault and alcohol use. Alcohol use could be used to cope with traumatic experiences such as sexual and physical dating violence (Kaukinen, 2014). However, research has also argued for a bidirectional relation between dating violence and alcohol use. That is, alcohol use can make one more vulnerable to experience dating violence (Spencer et al., 2020).

Second, our study focused on dating violence, harassment, and assault victimization, but did not assess perpetration. From the current study, no inferences can be made about sexual and gender identity-based differences in the perpetration of dating violence, harassment, and assault and how perpetration might be (bi-directionally) related to alcohol use. Studying perpetration among SGM adolescents might be relevant, considering that youth report victimization and perpetration at similar levels (Haynie et al., 2013; Martin-Storey et al., 2020). If within-group differences exist in the perpetration of dating violence, harassment and assault, this could be a potential avenue for future studies and a relevant topic to address in prevention and intervention programs.

Third, all dating violence, harassment, and assault variables were asked in the time frame of the past 12 months—the same time frame was not used for the questions on dating experiences. This means that participants who, for example, dated more than 12 months ago and experienced dating violence during this relationship did not report on their experiences with dating violence, which might have resulted in underreporting of experiences with dating violence.

Fourth, although this study acknowledged diversity in sexual and gender identity, it was not assessed how these identities might intersect with one another, as well as with other important identities such as race/ethnicity. Race/ethnicity might be especially relevant here considering that research shows that African-American youth are more likely to experience physical and verbal dating violence victimization compared to White youth (Haynie et al., 2013).

## Conclusion

This study explored the occurrence of sexual and physical dating violence and sexual harassment and assault among SGM adolescents considering the diversity of experiences with dating and sexual relationships. Findings point to the relevance of (sexual) violence experiences in and outside committed relationships in explaining sexual and gender identity-based differences in alcohol use.

## Supplemental Material

Supplemental material for this article is available online. Supplemental Material for Associations of Relationship Experiences, Dating Violence, Sexual Harassment, and Assault With Alcohol Use Among Sexual and Gender Minority Adolescents by W. J. Kiekens, L. Baams, J. N. Fish, R. J. Watson, in Journal of Interpersonal Violence

## Author Biographies

**W. J. Kiekens**, MSc, is a PhD candidate at the Department of Sociology and the Interuniversity Center for Social Science Theory and Methodology, University of Groningen, the Netherlands. His research interests include adolescent development and sexual orientation differences in health, well-being, and substance use.

**L. Baams**, PhD, works as an assistant professor at the Pedagogy and Educational Sciences Department at the University of Groningen. Overall, her research addresses health disparities among LGBTQ youth, and how these can be exacerbated or diminished by social environmental factors.

**J. N. Fish**, PhD, is an assistant professor in the Department of Family Science at the University of Maryland School of Public Health. Her research examines how youth experiences shape the health and wellness of LGBTQ people across the life course.

**R. J. Watson**, PhD, is an assistant professor in the Department of Human Development and Family Sciences at the University of Connecticut. His scholarship examines the health and well-being of sexual and gender minority young people.
