# Supplementary material for: Associations of Relationship Experiences, Dating Violence, Sexual Harassment, and Assault With Alcohol Use Among Sexual and Gender Minority Adolescents
Source: J Interpers Violence. 2021 Mar 14;37(17-18):NP15176–204. doi: 10.1177/08862605211001469 (PMC8917816; doi:10.1177/08862605211001469)
Supplement: Supplemental material for this article is available online. [file sj-pdf-1-JIV-10.1177_08862605211001469.pdf]

Table S1

Multinomial Regression Analyses with Sexual Identity Predicting Class Membership with bisexual as reference group

	Intermediate exposure to harassment and assault[a]		High exposure to dating violence and assault[a]		High exposure to dating violence, assault, and harassment[a]	
	Odds ratio	95% CI	Odds ratio	Odds ratio	95% CI	Odds ratio
Sexual identity						
Bisexual (ref)						
Gay or lesbian	1.12	[0.92, 1.32]	0.64***	[0.51, 0.77]	0.82*	[0.65, 1.00]
Heterosexual	0.96	[0.37, 1.56]	0.60*	[0.24, 0.96]	0.25	[0.02, 0.52]
Queer	0.93	[0.57, 1.29]	0.50***	[0.29, 0.71]	0.74	[0.43, 1.04]
Pansexual	1.34*	[1.03, 1.65]	0.87	[0.66, 1.09]	1.01	[0.74, 1.28]
Asexual	1.36	[0.91, 1.81]	0.58***	[0.34, 0.82]	0.99	[0.60, 1.37]
Questioning	1.01	[0.55, 1.47]	0.42***	[0.15, 0.68]	0.62*	[0.25, 0.98]
Other	1.26	[0.64, 0.96]	0.61*	[0.26, 0.96]	1.27	[0.66, 1.87]

Note. Controlled for gender identity race/ethnicity, region, age, and living situation

[a]Compared with the "Few dating experiences and low dating violence, assault, and harassment" class

* $p < .05$. ** $p < .01$. *** $p < .001$

Table S2

Multinomial Regression Analyses with Gender Identity Predicting Class Membership with cisgender girl as reference group

	Intermediate exposure to harassment and assault[a]		High exposure to dating violence and assault[a]		High exposure to dating violence, assault, and harassment[a]	
	Odds ratio	95% CI	Odds ratio	Odds ratio	95% CI	Odds ratio
Gender identity						
Cisgender girl (ref)						
Cisgende boy	0.93	[0.75, 1.11]	0.43***	[0.36, 0.50]	0.58***	[0.42, 0.74]
Transgender boy	1.00	[0.70, 1.30]	1.75**	[1.49, 2.01]	1.59*	[1.12, 2.06]
Transgender girl	1.17	[0.39, 1.94]	0.98	[0.54, 1.42]	1.77	[0.52, 3.01]
Non-binary/male at birth	1.08	[0.87, 1.29]	1.63***	[1.45, 1.80]	1.41**	[1.11, 1.71]
Non-binary/female at birth	1.14	[0.65, 1.63]	0.75	[0.48, 1.01]	0.92	[0.41, 1.42]

Note. Controlled for sexual identity race/ethnicity, region, age, and living situation. [a]Compared with the “Few dating experiences and low dating violence, assault, and harassment” class
* $p < .05$. ** $p < .01$. *** $p < .001$

Table S3

Multinomial Regression Analyses with Gender Identity Predicting Class Membership with transgender boy as reference group

	Intermediate exposure to harassment and assault[a]		High exposure to dating violence and assault[a]		High exposure to dating violence, assault, and harassment[a]	
	Odds ratio	95% CI	Odds ratio	Odds ratio	95% CI	Odds ratio
Gender identity						
Transgender boy (ref)						
Cisgender boy	0.93	0.63, 1.24]	0.24***	[0.15, 0.34]	0.36***	[0.23, 0.50]
Cisgender girl	1.00	[0.70, 1.30]	0.57***	[0.41, 0.73]	0.63***	[0.44, 0.81]
Transgender girl	1.17	[0.34, 1.99]	0.56	[0.08, 1.03]	1.11	[0.30, 1.93]
Non-binary/assigned male at birth	1.08	[0.74, 1.41]	0.93	[0.67, 1.18]	0.89	[0.61, 1.16]
Non-binary/assigned female at birth	1.15	[0.57, 1.72]	0.43***	[0.13, 0.73]	0.58*	[0.23, 0.92]

Note. Controlled for sexual identity race/ethnicity, region, age, and living situation.

[a]Compared with the " Few dating experiences and low dating violence, assault, and harassment " class

* *p* < .05. ** *p* < .01. *** *p* < .001

Table S4

Multinomial Regression Analyses with Gender Identity Predicting Class Membership with transgender girl as reference group

	Intermediate exposure to harassment and assault[a]		High exposure to dating violence and assault[a]		High exposure to dating violence, assault, and harassment[a]	
	Odds ratio	95% CI	Odds ratio	Odds ratio	95% CI	Odds ratio
Gender identity						
Transgender girl (ref)						
Cisgender boy	0.80	[0.26, 1.34]	0.44**	[0.05, 0.82]	0.33***	[0.09, 0.57]
Cisgender girl	0.86	[0.29, 1.43]	1.02	[0.17, 1.88]	0.57**	[0.17, 0.96]
Transgender boy	0.86	[0.25, 1.46]	1.79	[0.26, 3.32]	0.90	[0.24, 1.56]
Non-binary/assigned male at birth	0.93	[0.30, 1.55]	1.67	[0.27, 3.06]	0.80	[0.23, 1.36]
Non-binary/assigned female at birth	0.98	[0.22, 1.74]	0.76	[0.00, 1.57]	0.52*	[0.07, 0.97]

Note. Controlled for sexual identity race/ethnicity, region, age, and living situation. [a]Compared with the "Few dating experiences and low dating violence, assault, and harassment" class

* $p < .05$. ** $p < .01$. *** $p < .001$
